# Homologous versus Heterologous prime-boost COVID-19 Vaccination in autologous hematopoietic stem cell transplantation recipients: a blinded randomized controlled trial

**DOI:** 10.3389/fimmu.2023.1237916

**Published:** 2023-08-01

**Authors:** Leyla Sharifi Aliabadi, Manoochehr Karami, Maryam Barkhordar, Seyed Saeed Hashemi Nazari, Amir Kavousi, Mohammad Ahmadvand, Mohammad Vaezi

**Affiliations:** ^1^ Department of Epidemiology, School of Public Health and Safety, Shahid Beheshti University of Medical Sciences, Tehran, Iran; ^2^ Environmental and Occupational Hazards Control Research Center, Shahid Beheshti University of Medical Sciences, Tehran, Iran; ^3^ Cell Therapy and Hematopotic Stem Cell Transplantation Research Center, Research Institute for Oncology, Hematology and Cell Therapy, Tehran University of Medical Sciences, Tehran, Iran; ^4^ Hematology, Oncology and Stem Cell Transplantation Research Center, Tehran University of Medical Sciences, Tehran, Iran

**Keywords:** SARS-CoV-2, heterologous prime boost COVID-19 vaccination, hematopoietic stem cell transplantation, RBD subunit vaccine, inactivated vaccines, immunogenicity

## Abstract

**Background/Purpose:**

Optimizing vaccine efficacy is of particular concern in patients undergoing hematopoietic stem cell transplantation (HSCT), which mainly have an inadequate immune response to primary SARS-CoV-2 vaccination. This investigation aimed to explore the potential prime-boost COVID-19 vaccination strategies following autologous (auto-) HSCT.

**Methods:**

In a randomized clinical trial, patients who had already received two primary doses of receptor-binding domain (RBD) tetanus toxoid (TT) conjugated SARS-CoV-2 vaccine during three to nine months after auto-HSCT were randomized to receive either a homologous RBD-TT conjugated or heterologous inactivated booster dose four weeks after the primary vaccination course. The primary outcome was comparing the anti-S IgG Immune status ratio (ISR) four weeks after the heterologous versus homologous booster dose. The assessment of safety and reactogenicity adverse events was considered as the secondary outcome.

**Results:**

Sixty-one auto-HSCT recipients were recruited and randomly assigned to receive either homologous or heterologous booster doses four weeks after the primary vaccination course. The mean ISR was 3.40 (95% CI: 2.63- 4.16) before the booster dose with a 90.0% seropositive rate. The ISR raised to 5.12 (95% CI: 4.15- 6.08) with a 100% seropositive rate after heterologous (P= 0.0064) and to 3.42 (95% CI: 2.67- 4.17) with a 93.0% seropositivity after the homologous booster doses (P= 0.96). In addition, the heterologous group suffered more AEs following the booster dosage than the homologous group, but this difference was not statistically significant (p = 0.955). In multivariable analysis, the prime-boost vaccination strategy (heterologous versus homologous), the level of ISR before the booster dose, and the length of time between auto-HSCT and booster dose were the positive predictors of serologic response to a booster dose. No serious adverse event is attributed to booster vaccination.

**Conclusion:**

In patients who were primed with two SARS-CoV-2 vaccine doses during the first year after auto-HSCT, heterologous prime-boost COVID-19 vaccination with inactivated platform resulted in considerably enhanced serologic response and non-significantly higher reactogenicity adverse events than homologous RBD-TT conjugated prime-boost COVID-19 vaccination strategy.

## Background

In light of the recent coronavirus disease 2019 (COVID-19) outbreak, a serious medical crisis has been announced that is extremely harmful to patients with hematologic malignancies and those who have undergone hematopoietic stem cell transplantation (HSCT) (1, 2). Vaccination is a successful preventative strategy that can enhance immunity against COVID-19. However, inadequate immune responses to the two primary doses of SARS-CoV-2 immunization in HSCT patients compared to healthy people (3–5), along with waning immunity of primary vaccine doses, incited the World Health Organization (WHO) and other scientific bodies, including the European Society for Blood and Marrow Transplantation (EBMT), to advise additional or booster COVID-19 vaccines for immunocompromised patients and HSCT recipients (6, 7).

The approach of giving an additional COVID-19 vaccine as a homologous or heterologous prime-boost is an interesting object of research that has been addressed in several studies on healthy subjects so far (8–11). Therefore, there is a need to explore different prime-boost strategies for optimizing vaccine immunity in HSCT recipients.

Most of the reported data on SARS-CoV-2 vaccination in HSCT patients employed mRNA-based vaccines, while a minority used an adenovirus-based vector platform (3–5). The protein subunit platform, such as receptor-binding domain (RBD) components, is an alternative vaccine technology with advantages in safety and preservation conditions, becoming more cost-effective for low- and middle-income countries (12). Soberana 2, or PastoCovac, is a new SARS-CoV-2 recombinant RBD conjugated to tetanus toxoid (TT) that is licensed for emergency use in Cuba and Iran. In particular phase 1, 2, and 3 clinical trials, the safety and immunogenicity of Soberana 2 have been evaluated before (13–15).

A recent publication demonstrated that the serologic response to two primary doses of RBD-TT conjugated SARS-CoV-2 vaccine (PastoCovac) shortly after autologous HSCT (auto-HSCT) was effective but not as effective as in healthy controls (16); which is equivalent to the mRNA-based platform (4, 5). The present study aimed to compare the immunogenicity and safety of homologous than heterologous prime-boost vaccination regimens in auto-HSCT patients who received two primary doses of RBD-TT conjugated SARS-CoV-2 vaccine.

## Methods

### Trial design and participants

An investigator-initiated, participant and observer-blinded, randomized controlled trial was performed to compare the serological response following the homologous prime-boost vaccination using RBD-TT conjugated (PastoCovac, Pasteur Institute, Tehran, Iran) compared with the heterologous prime-boost using an inactivated platform (Vero Cell, Beijing Institute of Biological Products, China) in auto-HSCT recipients more than 18 years old who had received two primary doses of RBD-TT conjugated vaccine, independent of the pre-booster immunogenicity. Prior treatment with rituximab within six months, relapse of the underlying disease, previous allergy to the vaccine’s components, and history of SARS-CoV-2 infection following HSCT were the major exclusion criteria. The patients were recruited to study from Jul 22, 2022, to Jan 2, 2023, at the Hematology-Oncology-Stem Cell Transplantation Research Center (HORCSCT) of Tehran University, Tehran, Iran.

### Registration and ethical approval

The trial was registered with the Iranian Registry of Clinical Trial (IRCT20140818018842N24). It was approved by the research ethics committee of the HORCSCT (IR.TUMS.HORCST.REC.1401.005) and the School of Public Health and Neuroscience Research Center-Shahid Beheshti the University of Medical Science (IR.SBMU.PHNS.REC.1401.112). The study was conducted following the principle of the Declaration of Helsinki and Good Clinical Practice. All study participants provided written informed consent for the vaccine booster dose, blood samples to be collected, and results to be published.

### Randomization and blinding

Participants were randomly assigned to the homologous or heterologous arms in parallel with equal probability. The balanced block randomization list was generated using the block size of 4 and sample size through the research institute’s web-based software.

Trained vaccine administrators enrolled the participants and assigned them to the trial groups according to the randomization list. While maintaining confidentiality, the vaccine administrators loaded the vaccines in a separate cubicle and administered them in the vaccination room. All other team members were blinded to the randomization list, including those responsible for the safety evaluation, collecting information, data analysis, laboratory assessors, and participating patients.

### Intervention

Randomized patients received 0.5 mL of either homologous RBD-TT conjugated or heterologous inactivated COVID-19 booster dose injected intramuscularly into the deltoid area. All patients were visited before (at baseline) and four weeks ( ± 7 days) after the booster, and their blood samples were taken for immunogenicity assessment. All participants were also followed-up through weekly telenursing calls for safety monitoring, any occurrences of COVID-19, relapse of underlying disease, or cytopenia to Mar 21, 2023. Data from the case report forms (CRF) were entered and managed on electronic data capture forms developed on the web-based software of the institution.

We employed the ChemoBind SARS-CoV-2 Neutralizing Antibody Test Kit (ChemoBind, Tehran, Iran) to assess the overall antibodies targeting the receptor-binding domain (RBD) spike protein of SARS-CoV-2 through a semi-quantitative immunoassay. As per the manufacturer’s guidelines, an immune status ratio (ISR) for immunoglobulin G (IgG) below 0.8 indicates a negative result, while an ISR above 1.1 indicates a positive result. Ratios falling between these thresholds are inconclusive and require repetition.

### Outcomes

As a primary outcome, the anti-S serologic response at four weeks (± seven days) after the booster dose was compared between the two intervention groups by measuring IgG Immune status ratio (ISR), and positivity rate, defined as an increase in the ISR to the cut-off point for a positive result in the semiquantitative test, as described previously (16). Secondary endpoints included the incidence and severity of all reactogenicity adverse events up to 7 days after booster dose administration using the Common Terminology Criteria for Adverse Events (CTCAE) (grade 4-5) (17). All non-reactogenicity events, including laboratory-confirmed COVID-19, hospitalization due to SARS-CoV infection, and relapse of the underlying disease, were also explored as the secondary outcomes.

### Statistical analysis

The sample size of 29 per group would give 90% power to determine the significant difference in ISR between the two intervention groups, with a two-tailed test, 0.75 effect size, 0.05 significance level, and 1/1 allocation ratio, using Gpower software version 3.1.9.7.

In a randomized clinical trial, the intention to treat (ITT) is the primary analysis. Given that the study population included all randomly assigned patients who received the allocated booster dose, ITT and per protocol (PP) analyses will invariably produce identical results. Descriptive analysis was reported as mean with standard deviation (SD), median with interquartile range (IQR) for quantitative variables, and frequency with percent for qualitative variables. The variability of ISR was reported using a 95% Confidence interval (CI) around the point estimate.

The Chi-squared test was used to compare the distribution of a categorical variable. Adverse events were compared between groups using the Fisher exact test.

The linear regression approach was used to investigate the predictors of serologic response following the booster dose. In univariate analysis, predictors associated with higher ISR value (p ≤ 0.2) are incorporated into a multivariable model. All the tests were considered two-way, and a p-value < 0.05 was reported as statistically significant. All statistical analyses were conducted using STATA version 17 (StataCorp, LP, College Station, TX, USA). GraphPad Prism software, version 9.5.1 (GraphPad Software Inc., San Diego, CA, USA), was used for the graphical presentation.

## Results

### Participants

**Figure 1:**
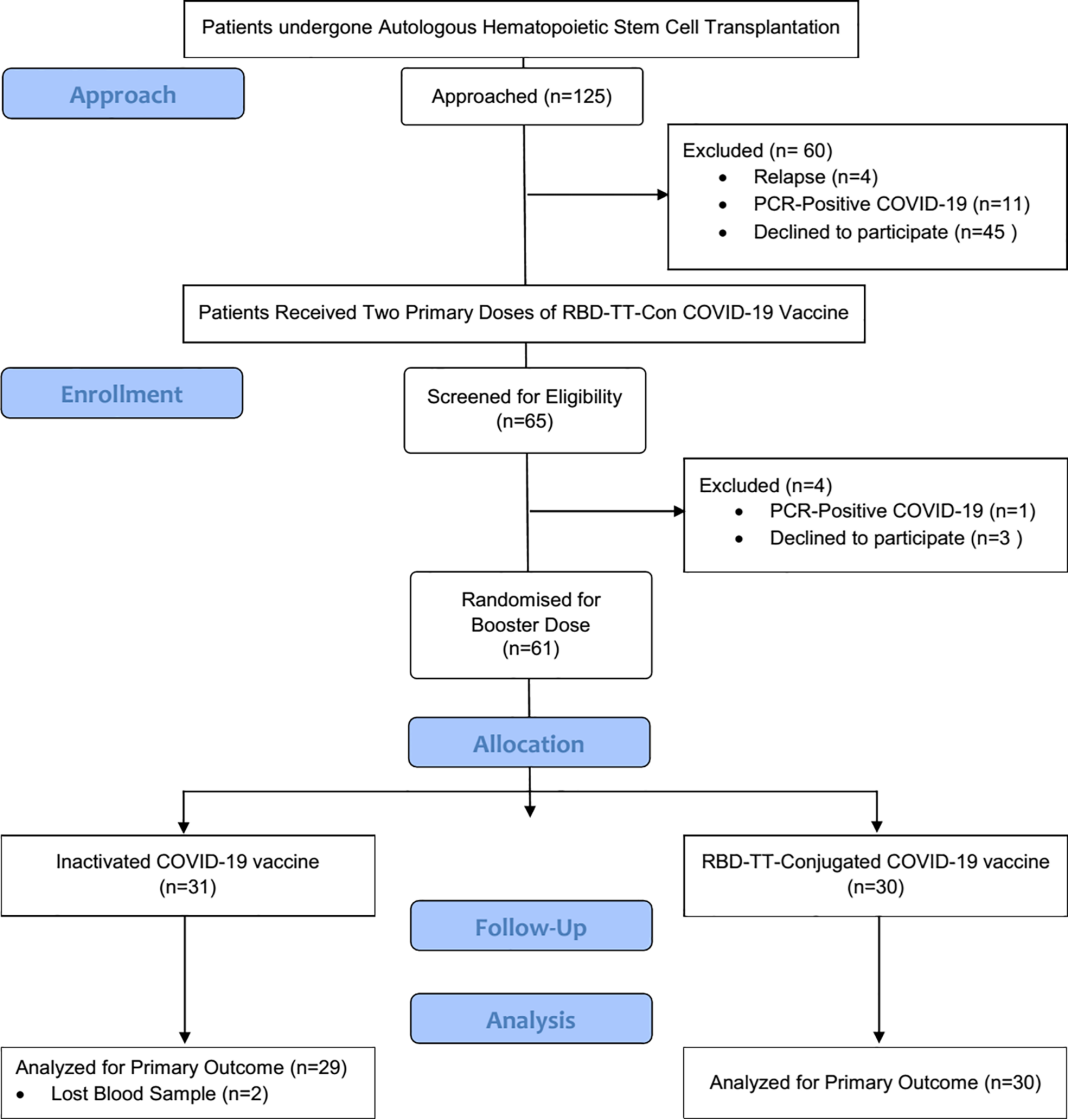
Flowchart of the randomized controlled trial. The chart depicts the subjects screened before the study, those recruited for Vaccination, and the processes for selecting or excluding patients.

The flow chart of study selection was presented in detail in **Figure 1**. Beginning in April 2022, one hundred and twenty-five auto-HSCT recipients were screened to get post-HSCT immunization. Of whom, 65 auto-HSCT recipients who had taken two primary doses of the RBD-TT-conjugated COVID-19 vaccine at a median time interval of 137 days following auto-HSCT were qualified to participate in the research. Three patients refused a booster dosage, and one was excluded due to PCR-Positive COVID-19. Sixty-one patients were recruited and randomly assigned to receive homologous or heterologous booster doses four weeks after the primary vaccination course.

**Table 1** lists the patients’ crucial baseline characteristics according to booster type. Following randomization, a homologous RBD-TT conjugated booster was given to 30 patients, including 12 women (40%) with a mean ± SD age of 51 ± 11 years, at the median (IQR) interval of 206 (178- 265) days after auto-HSCT. In the heterologous arm, at a median (IQR) interval of 219 (178- 277) days, 31 patients, including eight females (26%), with a mean ± SD age of 50 ± 11 years, got an inactivated vaccine.

**Table 1:** Baseline characteristics of participants based on the homologous and heterologous prime-boost COVID-19 vaccination in autologous hematopoietic stem cell transplant (auto-HSCT) recipients.

Characteristics		Booster Type	
		RBD-TT-Conjugated	Inactivated
Number of participants		30	31
Age		51 ( ± 11)	50 ( ± 11)
Sex	Female	12 (40%)	8 (26%)
Background Disease	Lymphoma	14 (47%)	12 (39%)
	MM	16 (53%)	19 (61%)
Pre-HSCT COVID-19 vaccination	Yes	25 (83%)	28 (90%)
Pre-HSCT PCR-positive COVID-19	Yes	17 (57%)	19 (61%)
ISR before the first vaccine dose		1.24 (0.70-2.50)	1.51 (1.20-2.87)
ISR before the booster dose		2.90 (1.58-5.60)	3.74 (1.79-4.55)
Time between HSCT and booster dose in days		206 (178-265)	219 (178-277)

Data are presented as frequency (%), mean (± standard deviation) and median (Q1- Q3)

RBD-TT-Conjugated, Receptor-Binding Domain (RBD)-Tetanus Toxoid (TT); ISR, Immune Status Ratio

HSCT, Hematopoietic Stem Cell Transplantation; MM, Multiple Myeloma

The indications for auto-HSCT in the RBD-TT conjugated and inactivated booster groups were multiple myeloma (MM) (53%, 61%) and lymphoma (47%, 39%). Before auto-HSCT, 83% of patients in the RBD-TT conjugated arm and 90% in the inactivated arm were completely immunized against SARS-CoV-2. Seventeen (57%) of the homologous arm and 19 (61%) of the heterologous arm had a history of PCR-positive COVID-19 before auto-HSCT. Nevertheless, there were no statistically significant differences between the two randomized groups’ baseline characteristics.

### Primary endpoint

Two of the thirty-one blood samples collected in the heterologous arm were lost. Moreover, the serological responses of 30 patients in the homologous arm and 29 patients in the heterologous arm were compared as the primary outcome.

**Figure 2:**
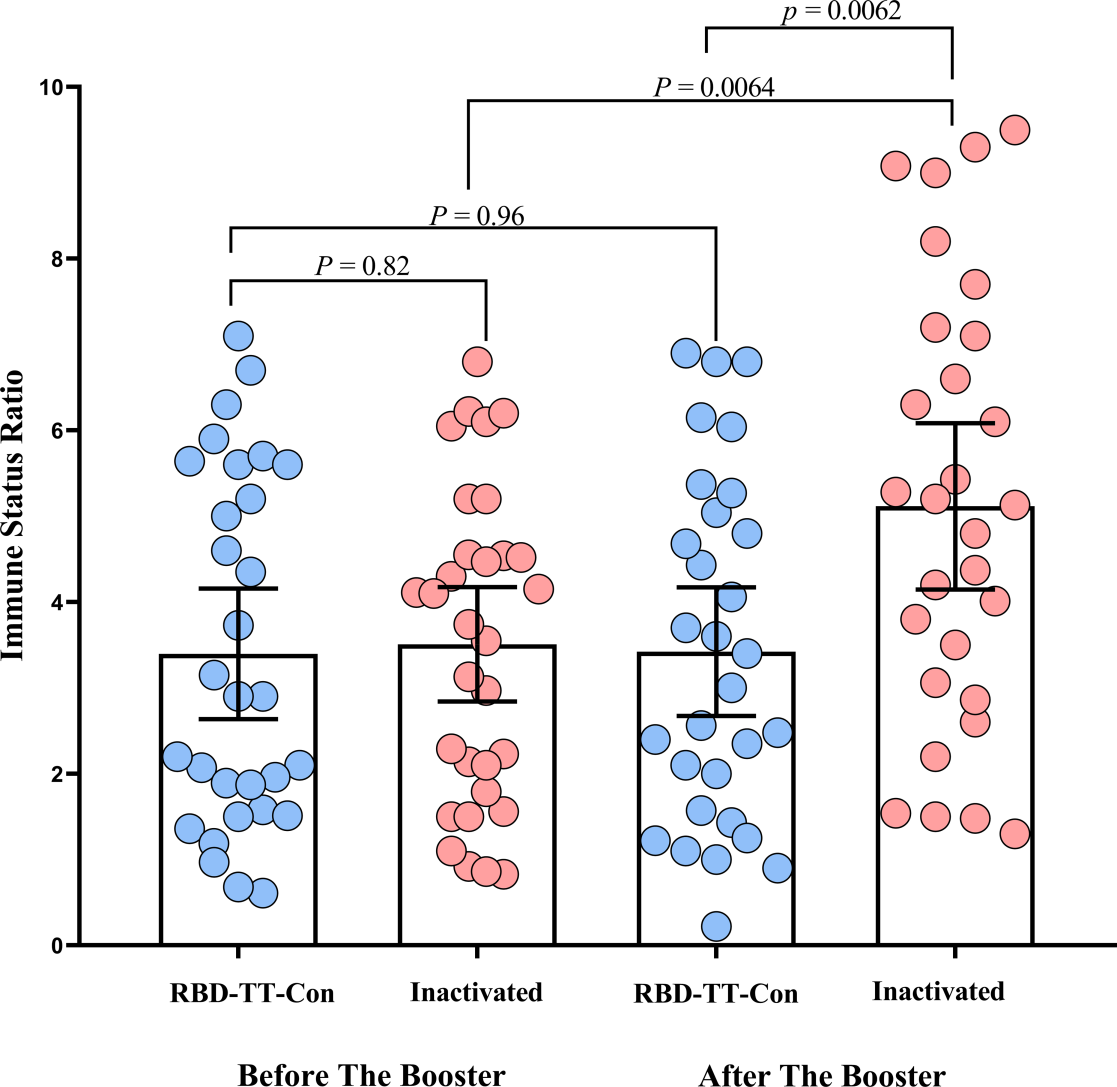
Scatter plot of the SARS-CoV-2 IgG immune status ratio in homologous and heterologous prime-boost arms before and after the booster dose in autologous hematopoietic stem cell transplant recipients.

**Figure 2** depicts a scatter plot of the SARS-CoV-2 IgG ISR in two intervention groups across two-time points before and following the booster dose. In the homologous arm, the mean (95% CI) ISR was 3.40 (95% CI: 2.63-4.16) before the booster dose, with a 90.2% seropositive rate; this increased to 3.42 (95% CI: 2.67-4.17) 4 weeks (± 7 days) after RBD-TT conjugated booster with a 93.0% seropositivity (P= 0.96). In contrast, in the heterologous arm, the mean (95% CI) ISR was 3.50 (95% CI: 2.84 - 4.17) before the booster dose, with a 90.0% seropositive rate, and raised significantly, reaching 5.12 (95% CI: 4.15 - 6.08) with a 100% seropositive rate four weeks (± 7 days) after the inactivated vaccine booster dose (P= 0.0064). Although, the pre-booster ISR values were equal between the two groups (P= 0.82).

### Secondary endpoints

**Figure 3:**
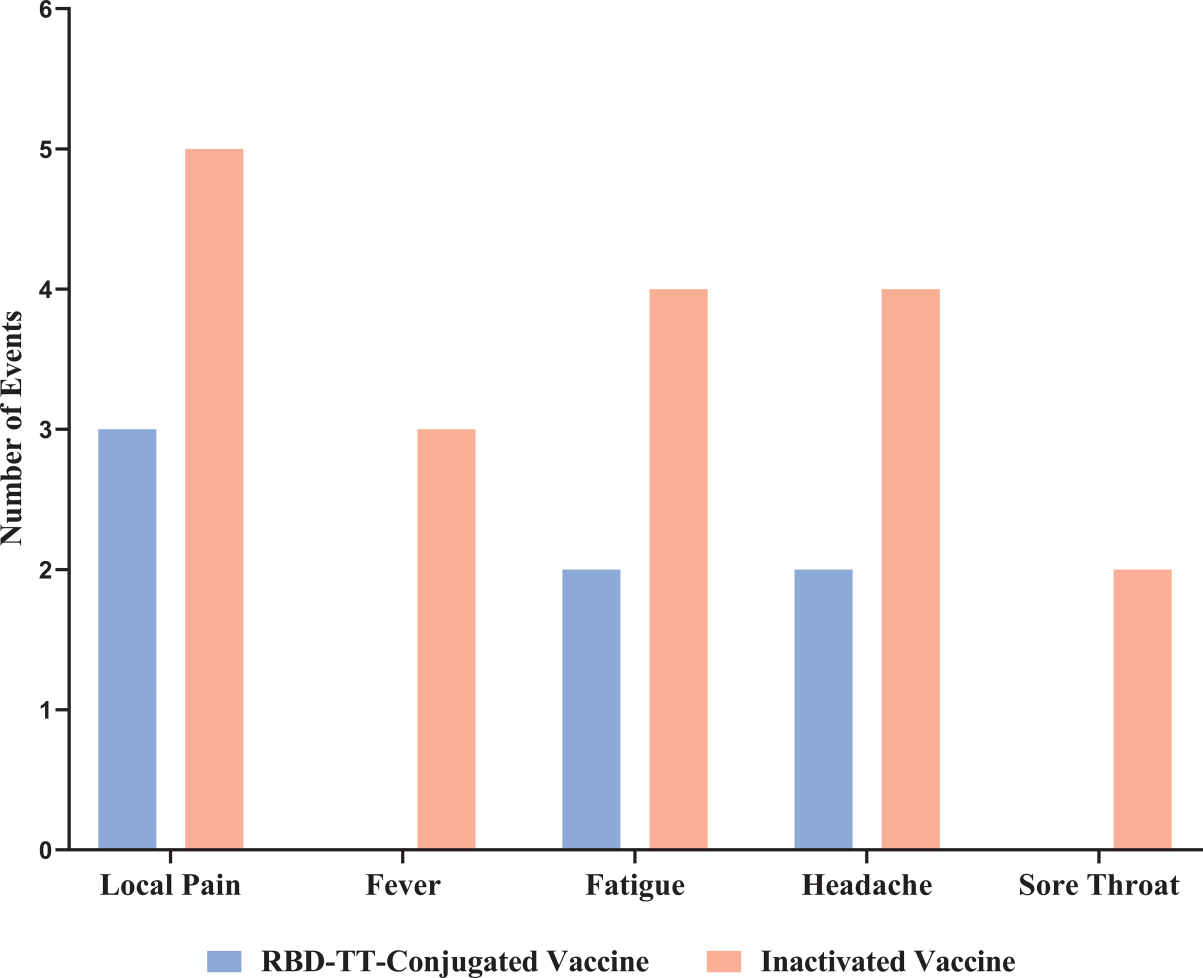
Reactogenicity adverse events up to 7 days after homologous and heterologous boosting in autologous hematopoietic stem cell transplant recipients.

Data on vaccine-related AEs are shown in **Figure 3**. The CTCAE shows no participant had grade 3 or 4 adverse events. The heterologous group suffered more AEs following the booster dosage than the homologous group, but this difference was not statistically significant (p = 0.955). Pain or tenderness at the injection site was reported by 5/31 (16%) of the heterologous and 3/30 (10%) of the homologous group. Four out of thirty-one (13%) of the heterologous arm experienced fatigue and a headache, as opposed to 2/30 (7%) of the homologous arm. Fever and sore throat were noticed only in three (10%) and two (6%) out of thirty-one heterologous arms, respectively.

At a mean follow-up of 171.95 (95% CI: 157.36 - 186.53) days after the booster dose, 2 of the 31 (6%) heterologous and 4 of the 30 (13%) homologous arm patients developed PCR-documented COVID-19 infections presented with mild respiratory symptoms; nonetheless, only one patient, from the homologous arm, was hospitalized. Through this, no recurrence of the underlying disorder appeared.

### Predictors of immune response

**Figure 4:**
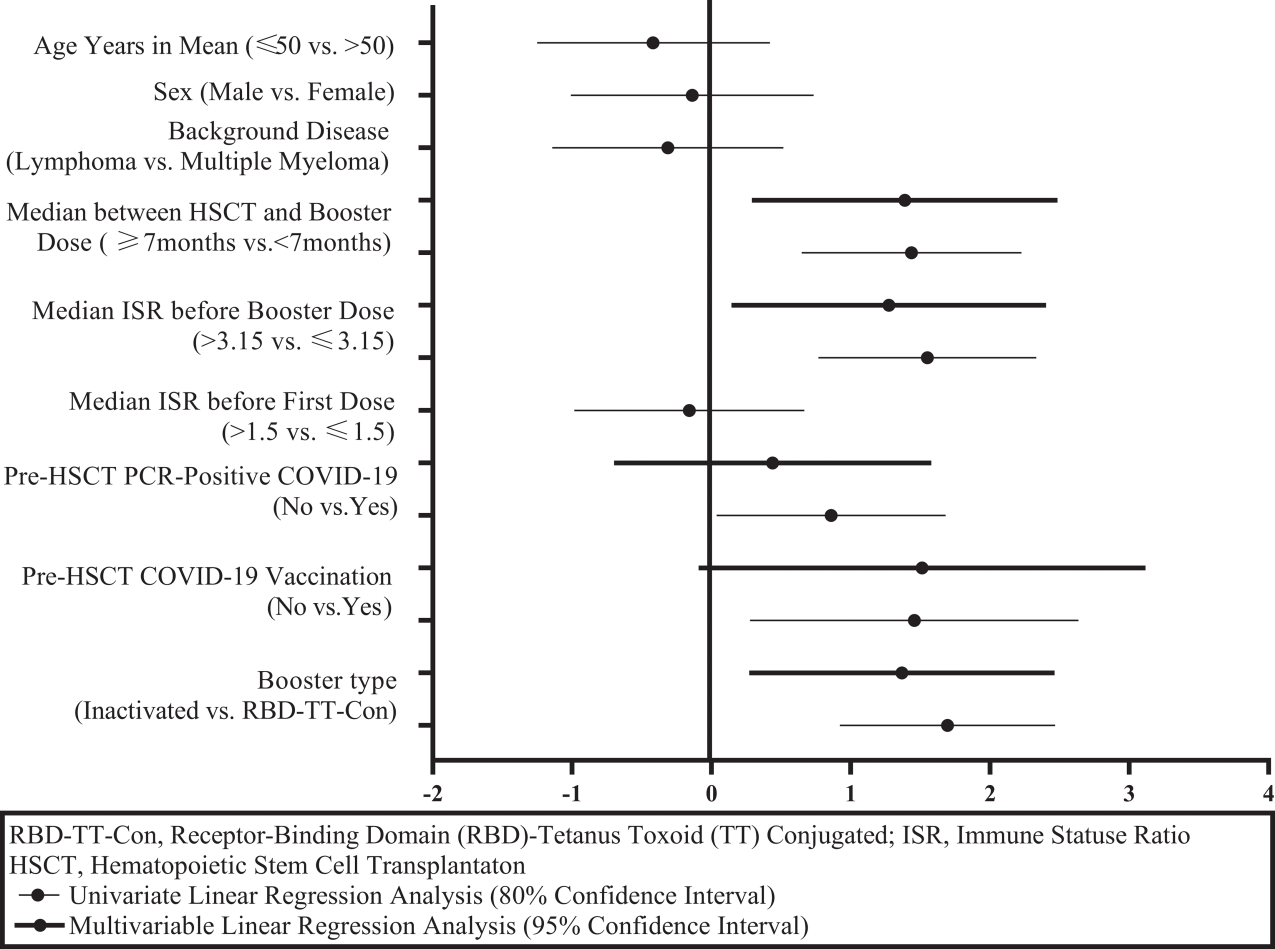
Graphical display of immunity predictors after a SARS-CoV-2 booster dose in autologous hematopoietic stem cell transplant recipients.

**Figure 4** and **Table 2** illustrate the univariate and multivariable linear regression analyses to identify the immune response predictors following the booster dose. In univariate analysis, pre-HSCT COVID-19 vaccination, pre-HSCT PCR-positive COVID-19, the baseline ISR before the booster dose, the time interval between HSCT and the booster dose, and the booster type (heterologous versus homologous) were associated with the higher immune response after the booster dose. However, in multivariable analysis, the baseline ISR before the booster dose (β= 1.27, 95% CI: 0.14- 2.40, P = 0.028), the time interval between HSCT and the booster dose (β= 1.39, 95% CI: 0.29- 2.48, P = 0.014), and the booster type (β= 1.37, 95% CI: 0.27- 2.46, P = 0.015) were still independent predictors of the immune response after the booster dose (adjusted R2 = 0.269).

**Table 2:** Univariate and multivariable linear regression analysis to determine the predictive factors of the immunogenicity following booster SARS-CoV-2 vaccine in auto-HSCT recipients.

Variable		Post-Booster ISR	Linear Regression Analysis for Post-Booster ISR			
			Univariate		Multivariable	
			Coefficients (B)	P value	Coefficients (B)	P value
**Age (Mean in Years)**	<=50	4.49 (2.55)	-0.42	0.515		
	>50	4.07 (2.34)				
**Sex**	Female	4.34 (2.64)	-0.14	0.839		
	Male	4.20 (2.33)				
**Background Disease**	MM	4.39 (2.52)	-0.31	0.626		
	Lymphoma	4.07 (2.32)				
**Pre-HSCT** **COVID-19 Vaccination**	Yes	4.45 (2.49)	1.45	0.115	1.51	0.064
	No	2.99 (1.42)				
**Pre-HSCT** **PCR-Positive COVID-19**	Yes	4.61 (2.48)	0.86	0.181	0.44	0.442
	No	3.75 (2.29)				
**Median ISR** **before First Dose Vaccine**	<=1.5	4.32 (2.57)	-0.16	0.805		
	>1.5	4.17 (2.28)				
**Median ISR** **before Booster Dose Vaccine**	<=3.15	3.51 (2.39)	1.55	0.013	1.27	**0.028**
	>3.15	5.06 (2.21)				
**Median Time** **between HSCT and Booster Dose**	<=7 months	3.57 (2.29)	1.43	0.022	1.39	**0.014**
	>7 months	5.00 (2.37)				
**Booster Type**	Inactivated	5.11 (2.54)	1.69	0.006	1.37	**0.015**
	RBD-TT-Con	3.42 (2.00)				

Data are presented as mean (standard deviation); ISR, Immune Status Ratio; HSCT, Hematopoietic Stem Cell transplantation

RBD-TT-Con, Receptor-Binding Domain (RBD)-Tetanus Toxoid (TT)-Conjugated.

The bold values shows the statistically significant P values.

## Discussion

The present study is the first trial comparing the homologous than heterologous prime-boost COVID-19 vaccination regimens throughout auto-HSCT recipients. In auto-HSCT patients who received two primary SARS-CoV-2 vaccines using RBD-TT conjugated platform, it was demonstrated that heterologous boosting with an inactivated platform yielded a superior serologic response and non-significantly more reactogenicity than homologous RBD-TT conjugated boosting. Exposure to various vaccine antigens could be attributed to the increased immunogenicity and reactogenicity identified following heterologous prim-boost COVID-19 immunization.

In numerous investigations on the general population in Brazil, Korea, and Chile, consistent results on improved immune response following the heterologous than homologous booster immunization among participants who received two primary doses of the COVID-19 vaccine have been shown (18–20). Likewise, in a randomized phase 4 study investigating the immunogenicity of four possible combinations of homologous and heterologous prim-boost with ChAdOx1 nCoV-19 (COVISHIELD™) and BBV152 (COVAXIN®), heterologous booster with ChAdOx1 nCoV-19 after BBV152 prime produced the greatest immune response (21).

Several studies have compared the immunogenicity of heterologous versus homologous booster vaccinations in solid organ transplants and immunocompromised patients (22–25). Consistent with study findings, Chiang et al. demonstrated superior serologic response in the heterologous boost with Ad26 compared to the homologous boost with the mRNA vaccine after two primary doses of mRNA in solid organ transplant recipients (22). On the contrary, Ji-Man Kang et al. (23) and Reindl-Schwaighofer et al. (24) noted no noticeable difference in humoral immunity after homologous and heterologous booster schedules in solid organ transplant recipients. In a blinded randomized controlled trial, Daniel Mrak et al. (25) reported that in immunosuppressed patients who failed to seroconvert after two mRNA vaccinations, the homologous booster with mRNA had a significantly higher seroconversion rate than the heterologous booster with vector vaccine.

Study findings demonstrated that both homologous and heterologous booster vaccinations exhibited reasonable reactogenicity AEs during the first week following administration, either mild or moderate. Although there were more reactogenicity AEs with heterologous than homologous boosters, the two arms had no significant difference. Numerous studies undertaken in the general population have revealed greater reactogenicity with heterologous than homologous prime-boost vaccinations, supporting the study findings (18, 21, 26). The Com-COV study’s findings (26), comparing four prime-boost combinations, demonstrated more systemic reactogenicity in heterologous than homologous prim-boost vaccination regimens.

Considering the predictors of immune response to booster doses, as shown in **Table 2** and **Figure 4**, it was determined that the booster type (heterologous versus homologous), a higher level of ISR before booster dose, and the length of time between auto-HSCT and booster vaccination were the independent predictors of a greater serologic response after booster vaccination. Consistent with the study findings, several previous studies proved that the time interval between HSCT and Vaccination predicts the magnitude of the serologic response to post-HSCT immunization (27, 28). Nevertheless, in a prior publication, no correlation between the interval from HSCT to the third dose of RBD-TT conjugated SARS-CoV-2 vaccine in allo-HSCT recipients was found (29), neither the interval from HSCT to the second dose of RBD-TT conjugated SARS-CoV-2 vaccine in auto-HSCT recipients (16).

The study’s limitations, advantages, and other relevant considerations are as follows. The present study could not determine the exact amount of anti-S antibodies using a semiquantitative serologic test. Since it was impossible to access a functional assay kit for SARS-CoV-2-specific T-cell responses, cellular immunity could not be evaluated. Regarding benefits, all patients received two primary doses of RBD-TT conjugated SARS-CoV-2 vaccine (16) and a homologous or heterologous booster dose in a constrained 3 to 12 months after auto-HSCT. This research encourages using a readily available and reasonably priced SARS-CoV-2 vaccine in a developing country, which may help optimize the vaccination strategy, particularly for special populations such as HSCT recipients.

In conclusion, this blinded, randomly assigned controlled clinical trial shows that both homologous and heterologous prime-boost COVID-19 vaccination strategies are immunogenic and safe in auto-HSCT recipients primed with RBD-TT conjugated early after HSCT. However, heterologous prime-boost COVID-19 vaccination with inactivated platform resulted in considerably enhanced immune response and insignificantly higher reactogenicity than homologous RBD-TT conjugated prime-boost.

## Data availability statement

The original contributions presented in the study are included in the article/supplementary material. Further inquiries can be directed to the corresponding author.

## Ethics statement

The studies involving human participants were reviewed and approved by the research ethics committee of the HORCSCT (IR.TUMS.HORCST.REC.1401.005) and the School of Public Health and Neuroscience Research Center-Shahid Beheshti the University of Medical Science (IR.SBMU.PHNS.REC.1401.112). The patients/participants provided their written informed consent to participate in this study.

## Author contributions

LS: Study concept and design; patient recruitment; randomization, data gathering, analysis of data, drafting of the manuscript; critical revision of the manuscript. MK: Study concept and design, critical revision of the manuscript. MB: Study concept and design; patient recruitment; data gathering, drafting of the manuscript; critical revision of the manuscript. SH: Confirming analysis of data, critical revision of the manuscript. AK: Confirming analysis of data, critical revision of the manuscript. MA: Performed antibody measurements and critical revision of the manuscript. MV: Critical revision of the manuscript. All authors contributed to the article and approved the submitted version.
